# ASPHER Statement for COP28. A Call for Action in Seven Points

**DOI:** 10.3389/phrs.2023.1606889

**Published:** 2023-12-27

**Authors:** Laurent Chambaud, Tara Chen, Chiara Cadeddu, Ana-Caterina Pinho-Gomes, Balázs Ádám, John Middleton, Susana Viegas, Nadav Davidovitch, Doris Zjalic, Flavia Pennisi, Lore Leighton, Robert Otok, Carlo Signorelli, The ASPHER Climate and Health Working Group

**Affiliations:** ^1^ Association of Schools of Public Health in the European Region (ASPHER), Brussels, Belgium; ^2^ Erasmus School of Health Policy & Management, Erasmus University Rotterdam, Rotterdam, Netherlands; ^3^ Institute for Global Health, University College London, London, United Kingdom; ^4^ Institute of Public Health, College of Medicine and Health Sciences, United Arab Emirates University, Al Ain, United Arab Emirates; ^5^ Faculty of Education, Health and Wellbeing, Wolverhampton University, Wolverhampton, United Kingdom; ^6^ NOVA National School of Public Health, Public Health Research Centre, Comprehensive Health Research Center, CHRC, NOVA University Lisbon, Lisbon, Portugal; ^7^ School of Public Health, Ben Gurion University of the Negev, Beer-Sheva, Israel; ^8^ Department of Life Sciences and Public Health, Università Cattolica del Sacro Cuore, Rome, Italy; ^9^ School of Public Health (Igiene e medicina preventiva), University Vita Salute San Raffaele, Milan, Italy

**Keywords:** climate change, health, COP28, ASPHER, Schools of Public Health

**Statement of the Association of Schools of Public Health in the European Region (ASPHER)**

With this statement, issued in the context of COP28, the Association of Schools of Public Health in the European Region (ASPHER) calls for immediate action on Climate Change to protect the Health of human populations and ecosystems and, by so doing, preserve the future of our planet.

The impact of climate change on health is an urgent and demanding public health emergency. It is the single biggest threat to global health, peace, and security, a crisis multiplier, and a significant driver of health inequalities [1]. Continued disagreement and procrastination will only exacerbate this planetary challenge as it is well documented [2] that populations are already living with the effects and suffering the health consequences of global climate disruption. The imperative to globally address climate change is met with varying degrees of commitment among nations, as elucidated by the Climate Change Performance Index 2023 (CCPI) [3]. The European Union (EU) is a notable contributor, demonstrating commitment through a comprehensive legislative framework.

While sharing in the broader World Health Organization (WHO) call for the necessary phase out of fossil energy, the shift to a greener society and biodiversity conservation [4], our member community of Schools of Public Health draw policymakers’ and citizens’ attention to seven specific requirements to reduce the impacts of the effects of climate change and protect public health:

**1. Recognition of the linkage between Climate Change and Health.**

As a network of institutions concerned with public health education and training, ASPHER welcomes the first full day dedicated to Health in this COP28 and we aim to be at the negotiating table in the future to make health an indicator of progress in fighting climate change. This is a necessary step to remedy a lack of prior high-level dialogue recognizing the vital interconnection of Climate Change and Health. We have to strengthen this link [5], as an increasing number of studies confirm the effects of climate change, and more broadly of environmental degradation, on health. At the European level, the role of the new European Climate and Health Observatory^1^ is critical. ASPHER, as a partner, is providing its expertise to this Observatory.

**2. An integrated global approach to Climate Change and Health.**

The impact of climate change on health is a critical global concern that demands robust scientific evidence and a comprehensive viewpoint, incorporating One Health, EcoHealth and Planetary Health perspectives when approaching the problem [6, 7]. We need to look at direct and indirect effects of Climate Change on Health, also taking into account the global effect of environmental degradation due to human activities (e.g., pollution, biodiversity loss, water mismanagement).

**3. Solidarity across countries to reduce greenhouse gas emissions while reducing the impacts of Climate Change on Health.**

Health organisations and systems will need the resources to practice sustainable healthcare, contributing to the reduction of greenhouse gas emissions in adherence to nationally determined contributions (NDCs) under the Paris Agreement without compromising other environmental impacts, while also preparing for the challenges due to the novel health needs in rapid transformation from climate change despite a paucity of health professionals [8, 9]. Solidarity is required between countries with different levels of societal development and resources. Leadership will be needed to plan for and limit greenhouse gas emissions of healthcare systems with the healthcare sector currently responsible for almost 5% of global greenhouse gas emissions, while allowing for development of resources and activities in health. This means prioritizing health promotion, disease prevention and primary care. Contributions of new and frugal health technologies may also help in achieving this transformation.

**4. Reducing the impact of Climate Change on Health inequalities.**

Well-documented health inequalities will increase with the effects of climate change. The COVID-19 pandemic has shown how planetary crises have a dramatic effect on health inequalities. These threats could be called syndemic and call for an account of not only clinical, but also biological and social interactions [10]. Our member schools community advocates for an explicit recognition during COP28 of the urgent need to fight for climate justice.

**5. Training and capacity building in Climate Change and Health for multiple stakeholders.**

Training activities in Climate Change and Health must shift into high gear—not only in core curriculum for academic programs, but also reinforced capacity building and life-long learning as a critical area of competence for a prepared workforce [11]. It is also necessary for public health agencies to be fully trained, properly resourced, and active in preparedness to reduce the impacts of climate change on health [12].

Schools of Public Health are the perfect entities to enlarge the vision of training, not only for public health and health professionals, but also many other professions (e.g., urban planners, social workers, teachers, lawyers, journalists, engineers), politicians, activists, NGOs and other stakeholders. COP28 must recognize the urgent need to develop a common set of training in all countries and regions. As a member of the Global Consortium of Climate Health Education (GCCHE),^2^ ASPHER participates in GCCHE’s mapping of trainings given by Schools of Public Health around the world and in developing specific courses. ASPHER as a member of the Agency for Public Health Education Accreditation (APHEA)^3^ will seek for formal accreditation of Climate Change and Health training and educational events through APHEA.

**6. Well-funded interventional transdisciplinary research on Climate Change and Health.**

A concerted effort must be undertaken in transdisciplinary research, at national and international levels. A particular attention must be paid to interventional research, involving civil society. While further research cannot be held as an excuse for delay, we have to continue to document not only the direct and indirect effects of Climate Change on Health, but also to evaluate the effectiveness of programs and actions designed to eliminate or reduce the impact of these effects, in order to define efficient interventions, and to adapt and prepare for changes that will take place. We pledge for a global fund dedicated to such research as well as a dedicated network to facilitate communication and research between different disciplines, in order to cross disciplinary borders to find new solutions and paradigms.

**7. Advocacy on Climate Change and Health.**

Advocacy is required at all levels—local, national, regional, international and global—to raise awareness, build preparedness, influence political decisions and secure adequate funding to meet the challenges of climate change and its effects on health. Schools of Public Health are involved and will reinforce their advocacy and awareness activities. A large alliance is necessary, to effectively facilitate advocacy based on a wide spectrum of professional knowledge and practice. Professionals will require tools to bring about change such as the Climate Change Litigation toolkit [13] ASPHER will develop and participate in scientific and academic networks [e.g., as a member of the Global Network for Academic Public Health (GNAPH)^4^ and partner with the European Public Health Association (EUPHA)^5^], as well as build and support broader coalitions in order to underline political and public awareness [e.g., as a member of the European Public Health Alliance (EPHA)^6^ and partner with the International Association of Public Health Institutes (IANPHI)^7^].

ASPHER has pledged itself to these seven points on Climate Change and Health and is actively pursuing them through the actions of the ASPHER Climate and Health Task Force as well as the broader initiatives of the Association. We are driven by mounting evidence of the climate change and health crisis and the impacts it has on our planet, ecosystems and current and future human generations. Under the auspices of COP28, we challenge all of you to join us!

## Footnotes

1
www.climate-adapt.eea.europa.eu/en/observatory/

2
https://www.publichealth.columbia.edu/research/programs/global-consortium-climate-health-education

3
www.aphea.be

4
www.globalnetworkpublichealth.org

5
www.eupha.org

6
www.epha.org

7
www.ianphi.org

## Editorial Note

The authors are responsible and liable for the contents of this Society Statement. Society Statements in PHR are checked for clarity and approved by an Editor-in-Chief. Society Statements are not externally peer reviewed.
